# Axial Growth Driven by Physical Development and Myopia among Children: A Two Year Cohort Study

**DOI:** 10.3390/jcm11133642

**Published:** 2022-06-23

**Authors:** Shida Chen, Yangfeng Guo, Xiaotong Han, Xinping Yu, Qianyun Chen, Decai Wang, Xiang Chen, Ling Jin, Jason Ha, Yuting Li, Yabin Qu, Rong Lin, Mingguang He, Yangfa Zeng, Yizhi Liu

**Affiliations:** 1 State Key Laboratory of Ophthalmology, Zhongshan Ophthalmic Center, Sun Yat-sen University, Guangdong Provincial Key Laboratory of Ophthalmology and Visual Science, Guangzhou 510060, China; chenshd3@mail.sysu.edu.cn (S.C.); lh.201205@aliyun.com (X.H.); yu-xinping@163.com (X.Y.); torunchen@163.com (Q.C.); wangdecai@gzzoc.com (D.W.); chen1094@hotmail.com (X.C.); lingjin41@yahoo.com (L.J.); liyuting3@mail.sysu.edu.cn (Y.L.); mingguang_he@yahoo.com (M.H.); 2 Health Promotion Center for Primary and Secondary Schools of Guangzhou Municipality, Guangzhou 510180, China; guoyangfeng2021@126.com; 3 Centre for Eye Research Australia, East Melbourne, Melbourne, VIC 3002, Australia; jason.ha1@unimelb.edu.au; 4 Royal Victorian Eye and Ear Hospital, East Melbourne, Melbourne, VIC 3002, Australia; 5 Guangdong Provincial Center for Disease Control and Prevention, Guangzhou 511430, China; yabinqu@163.com; 6 School Health Unit, Guangzhou Center for Disease Control and Prevention, Guangzhou 510440, China; linr@gz.gov.cn; 7 Centre for Eye Research Australia, University of Melbourne, Melbourne, VIC 3002, Australia; 8 Centre for Eye Research Australia, Royal Victorian Eye and Ear Hospital, Melbourne, VIC 3002, Australia

**Keywords:** myopia, axial length, children, physical development

## Abstract

Background: The physical process of axial length growth among children and its role in the occurrence of myopia remain insufficiently explored. In this study, we investigate the patterns of ocular axial growth among persistent myopia (PM) and persistent non-myopia (PNM) children aged 3 to 15 years. Methods: A group of 6353 children aged 3 to 15 years, selected from rural schools in China, were followed up annually for 2 years. Biometric measurements including axial length (AL) and spherical equivalent refraction (SER) were obtained. Body height was recorded. Children were divided into two groups: PM group defined as SER of −0.50 D or less; PNM group defined as −0.50 D < SER < +3.0 D during follow-up. Results: Annual AL growth was fairly consistent for PNM eyes of children aged 3 to 11 years and then reduced significantly (independent *t* test, *p* < 0.001) for children aged 12 years and older. This pattern of AL changes was similar for PM children, although the AL growth was greater among them. Among children aged 6 and older, body height change was concomitant to AL growth (*p* < 0.01) and SER myopic shift (*p* < 0.001) until reaching 12 years old (*p* = 0.308 and *p* = 0.679, respectively). Conclusions: Stature growth and AL growth are both remarkable and consistent and concomitant but start to attenuate when the children reach 10 to 12 years old among emmetropic children. This observation suggests that AL growth is driven by physical development until 12 years old, whereas its excessive growth is dominated by myopia development.

## 1. Introduction

The process of ocular axial length (AL) growth varies at different ages of development, with the maximal growth occurring prior to 2 years old [1,2], which tapers off by 3–6 years of age (approximately 0.14 mm/year) [3]. It has been considered that at the early stage of human life, axial length is driven by emmetropization, a physiological process of refractive development from hyperopia to emmetropia [4,5], and after this, excessive AL elongation is exhibited by those individuals who develop myopia, and AL can increase considerably until young adulthood [6].

Physical development, as a normal process of human development, is exhibited by increases in stature and ocular size as determined by age, gender and genetic background. On the other hand, myopia develops mainly during childhood, resulting from excessive elongation of the eye, which leads to images of distant objects focused in front of the retina [4]. Recent studies have also raised the possibility of a mechanism of AL growth secondary to myopia development that is often driven by environmental factors, such as increased educational intensity, near work and urbanization [7,8,9,10]. These two factors are substantially different, as physical development is driven by genetic factors and therefore is more linear and predictable, and myopia development as determined by environmental factors tends to be more varied. Some cross-sectional studies and longitudinal studies have demonstrated that AL elongation is closely associated with the development of body height [8,11] and further propose that there might be some common pathway for the development of eye size and body size in children; see refs. [12,13,14]. However, whether axial length is driven by the two factors, and clearly disentangling the two factors and characterizing their individual and synergistic effects on AL elongation, remain to be explored.

In this study, we try to disentangle the two factors that play a key role in AL growth, including physical development, which is reflected as body height and myopia development as displayed by SER. Therefore, we set up a cohort study in the semi-rural areas in Guangzhou, China, where the children are much less impacted by myopigenic environmental factors and therefore the prevalence of myopia is substantially lower, and we report the report the longitudinal changes in AL, spherical equivalent and height among persistent myopia (PM) and persistent non-myopia (PNM) children aged 3 to 15 years.

## 2. Materials and Methods

### 2.1. Study Population

This is a prospective longitudinal study with a total of 6353 children aged 3 to 15 years, recruited from two administrative districts (Zengcheng and Huadu) of Guangzhou, China, and followed up annually for 2 years. All the children were divided into four groups: (1) 1433 participants at first-year kindergarten level (Grade 0, G0); (2) 1561 participants from first year (Grade 1, G1); (3) 2671 participants from fourth year (Grade 4, G4) of primary school; (4) 1385 participants from the first year of junior high school (Grade 7, G7). All the participants from G0, G1 and parts of G4 were from Zengcheng district, and the rest from Huadu district. Written informed consent was obtained from parents and legal guardians, after the study rationale and procedures were explained in detail to them during a school seminar. This study conformed to the tenets of the Declaration of Helsinki and obtained ethical approval from the Institutional Review Board of Zhongshan Ophthalmic Center (2018KYPJ079). The study was registered on clinicaltrials.gov (NCT03589937).

The spherical equivalent refraction (SER) was calculated as the sum of the spherical power (D) and half of the cylinder power (D). Only data from the right eye were used, since there was a high correlation between the right and left eye in the same individual. Myopia was defined as an SER of −0.50 D or less; persistent myopia (PM) was defined as an SER of −0.50 D or less during the two-year follow up; persistent non-myopia (PNM) was defined as −0.50 D < SER < +3.0 D during the two-year follow-up. In this study, we included the entire PM and PNM groups, but excluded children who displayed unsuccessful cycloplegia, those with unsuccessful biometric measurements and children with abnormal ocular anterior and posterior segment findings, or astigmatism greater than 5.0 D in cylinder or high myopia (SER ≤ −6.0 D).

### 2.2. Examinations and Measurement

Ocular examinations were conducted annually during school days from 2018 to 2020. Ocular biometry was measured before pupil dilation with non-contact partial coherence laser interferometry (IOL Master; Carl Zeiss Meditec, Oberkochen, Germany). Five separate measurements were averaged for AL. Cycloplegia was achieved with two drops of 1% cyclopentolate, administered 5 min apart, with a third drop administered after 20 min. Cycloplegia was considered complete if the pupillary light reflex was absent and the pupil was dilated to at least 6 mm. Cycloplegic autorefraction (KR8800, Topcon, Tokyo, Japan) was performed on all participants, with the average of three readings obtained for each eye. The anterior segment, including the eyelid, conjunctiva, cornea, iris and pupil, and the posterior segment, including the fundus, optic disc and macula, were then evaluated with slit-lamp examination and indirect ophthalmoscopy, performed by an ophthalmologist. Height was measured to the nearest 0.1cm with a height measure (SUHONG, Changzhou, China), with the participants standing without shoes.

### 2.3. Statistical Analysis

Data from the right eyes of the participants were used. The baseline distributions of the participants’ characteristics, including age, SER and AL, were reported with means (standard deviation, SD) or medians (interquartile range, IQR), depending on whether or not the normality was satisfied, respectively. The normality of the continuous data was checked using the Kolmogorov–Smirnov normality test and a histogram. A longitudinal change in the distributions of AL and SER was described with a box plot. The prevalence of myopia and 95% confidence intervals were calculated for each grade and sex at each time point. A Lowess plot with a smoothing value of 0.50 was drawn to show AL and SER changes from 3 to 15 years of age. The mean rate of change during the two-year follow-up for the ocular characteristics, including SER, AL and body height for each of the grades, was calculated. Body height was divided into tertiles and the AL trend across height levels by age was tested among PNM participants. The trend of the prevalence of myopia through height levels and the predicted probability of myopia incidence during two follow-up years were illustrated by a histogram and logistic probability plot separately. Lowess plots for AL and SER change across age by height tertiles were constructed for PNM participants. Linear regression models were fitted to assess the association of height increase with SER shift and AL growth, respectively. A two-sided *p* value of 0.05 or less was considered statistically significant. Lowess plots were created using SAS 9.4 (SAS Institute Inc., Cary, NC, USA) and all other analyses were performed using Stata 16.0 (StataCorp, College Station, TX, USA).

## 3. Results

A total of 7312 children were eligible for this study, and 262 (3.58%) of them declined to take part in the study. Of the enrolled 7050 participants, 697 were excluded: 2 children with unsuccessful cycloplegia, 94 participants with unsuccessful biometric measurements and 601 participants with abnormal ocular anterior and posterior segment findings or astigmatism greater than 5.0 D in cylinder or high myopia (SER ≤ −6.0D), leaving a total of 6353 (90.1%) participants for the final analysis (Supplementary Figure S1 and Table S1). At baseline, there was no statistically significant gender difference (*p* = 0.608) among each enrolled grade from G0 to G7. Boys had a longer axial length than girls; however, there were more myopic eyes among girls. The detailed baseline characteristics are shown in Supplementary Table S2.

**Figure 1:**
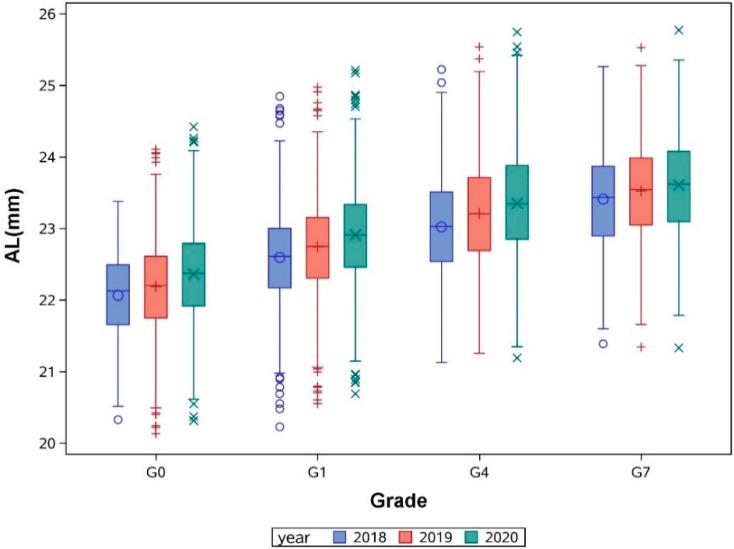
Annual axial length (AL) change from 3 to 15 years old in children from different grades with two-year follow-up.

We divided these children into two groups: a PM group defined as an SER of −0.50 D or less, and a PNM group defined as −0.50 D < SER < +3.0 D during follow-up. The axial length was much shorter in the PNM group than the PM group for each grade (with all *p* < 0.001, Table 1), and the axial length gradually increased in each grade with the two-year follow-up (Figure 1).

**Figure 2:**
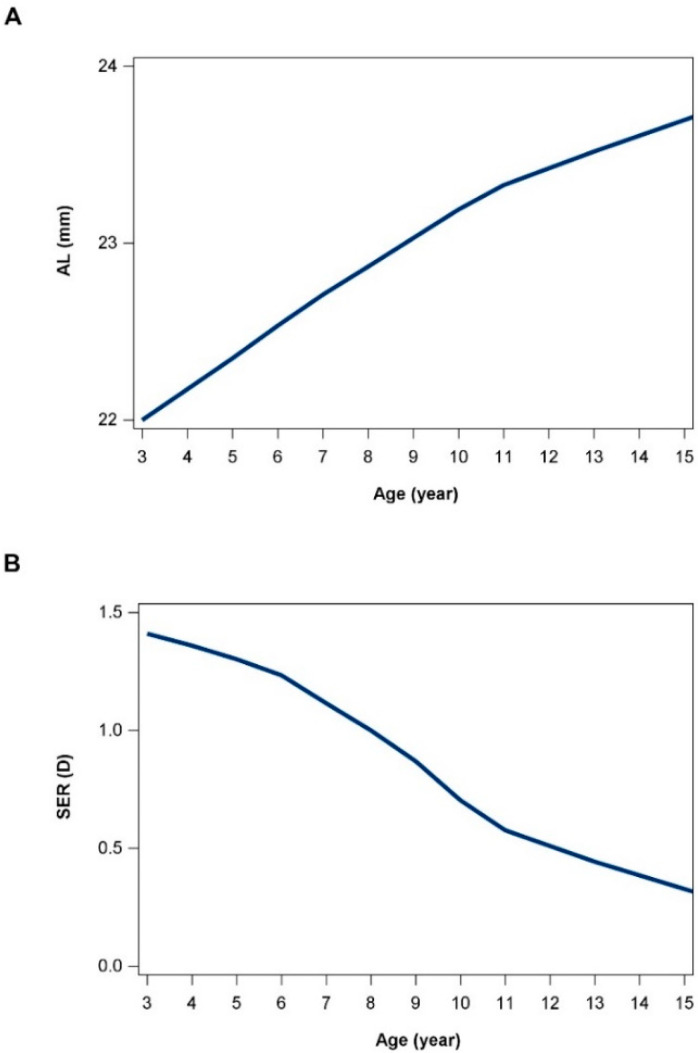
Axial length (AL) and spherical equivalent refraction (SER) change from 3 to 15 years of age. (**A**) The change in AL. (**B**) The change in SER. Data were plotted pooling all the participants with two-year follow-up. The current data were interpreted as longitudinal data with minimal cohort effect.

The mean annual elongation of axial length in PNM children was +0.18 (0.09) mm for G0 aged from 3 to 6 years old, +0.16 (0.08) mm for G1 aged from 6 to 9 years old and +0.17 (0.09) mm for G4 aged from 9 to 12 years old, which decreased to +0.11 (0.08) mm after 12 years of age. Similarly, after 12 years old, the mean annual change rate of SER decreased from −0.24 (0.21) D to −0.12 (0.22) D in PNM children (Table 2). For PM children, the elongation of axial length had a similar trend; however, there was a faster elongation rate across all ages, with +0.21 (0.11) mm AL elongation even after 12 years old, corresponding to an annual SER change of −0.42 (0.26) D (Table 2). We pooled all the participants with two-year follow-up, and the data were interpreted as longitudinal data with a minimal cohort effect, as shown in Figure 2. For PNM children, the mean (SD) axial length gradually increased from 22.07 (0.58) mm to 23.60 (0.72) mm from age 3 to 15 years old, corresponding to a gradual decrease in SER; both had a turning point around 10 to 12 years old, after which the growth rate was attenuated, with an annual AL growth of 0.19 (0.07) mm, and it then reduced significantly to 0.11 (0.07) after the age of 12 years (independent *t* test, *p* < 0.001).

**Figure 3:**
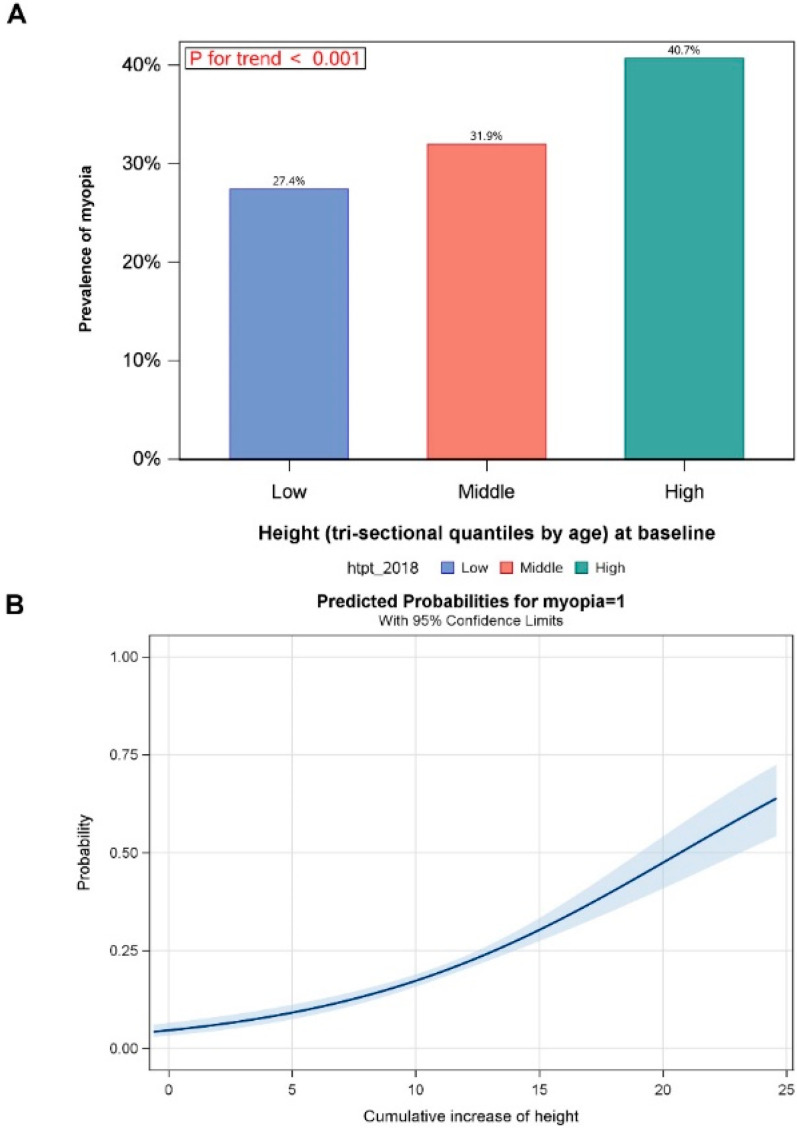
The effect of body height on the prevalence of myopia. (**A**) Different prevalence of myopia in height change in tertiles from 3 to 15 years of age; higher body height corresponds to higher prevalence of myopia (*p* < 0.001). (**B**) Higher cumulative increase in height corresponds to higher probability of myopia occurrence with two-year follow-up.

The mean annual growth rate of body height was around 4 cm to 6 cm, with no statistically significant difference between PNM and PM children (both *p* > 0.05) (Table 2). Multiple linear regression models were fitted to explore the associations among SER, axial length and body height change after adjusting for age and gender; before G7, the growth in body height was closely related to the change in AL; however, the growth in body height had a limited effect on the change in SER (Table 3). Greater body height corresponds to a higher prevalence of myopia (*p* < 0.001, Figure 3A). With the two-year follow-up, a higher cumulative increase in height corresponded to a higher probability of myopia occurrence (Figure 3B). For children with persistent non-myopia, higher body height corresponds to longer AL, before 12 years old (Supplementary Table S3 and Figure S2A). However, there was no relation between body height growth and change in SER (Supplementary Figure S2B).

## 4. Discussion

This is among the first studies to report the longitudinal change in axial length separately for PNM and PM groups of children aged 3 to 15 years in a Chinese population in a semi-rural area with a low prevalence of myopia. AL growth increases from age 3, until 10 to 12 years of age, at which point growth begins to attenuate and then stabilize. This pattern of AL growth is concomitant with stature growth and similar among PNM and myopic children, although the amount of growth is greater in myopic children. This observation suggests that AL growth is in fact driven by physical development until 10 to 12 years old, and excessive AL growth is augmented by processes of myopia development, which can occur at any age.

Based on previous studies including children and adults (the Reykjavik Eye Study of Adults [15], the Sydney Myopia Study of Australian children [16], Singaporean studies in children and adults [17,18] and the Anyang University Students Eye Study [19]), taller persons have longer ALs, and also AL is closely associated with body height. For every 10 cm difference in body height, there was an approximately +0.23 mm longer AL for adults [18], +0.29 mm longer AL for boys and +0.32 mm longer AL for girls [17]. Another cross-sectional study demonstrated that the cessation age for AL and stature growth is in fact very similar, although AL growth may persist after the period of usual termination of physiological growth for myopic eyes [20]. Furthermore, other studies have observed that taller, younger individuals tend to have a longer AL and are more myopic, which has led to speculation that this may be in fact mediated by improved nutrition during the growth period of each successive generation [11]. The Guangzhou Twin Registry study demonstrated that the mean annual increases in AL and height were 0.22 ± 0.17 mm and 3.93 ± 3.02 cm, respectively, in children aged from 7 to 15 years, with longer ALs correlated with each 10 cm difference in body height [12]. They also found that around 89% of the phenotypic correlation between AL and height was found to be due to shared genetic factors [21]. In our cohort of children aged 3 to 15 years, every 10 cm increase in body height corresponds to 0.2 mm AL elongation for non-myopic eyes, but when the child reaches 10–12 years old, both the AL growth and body height growth slow down. This association between AL and stature growth remains statistically significant after adjusting for age and sex. This observation confirms that ocular size development is concomitant with physical development, both of which may share common pathways.

The temporal progression of myopia development overlaps the physiological eye growth, which results in and maintains emmetropia [13]. Therefore, it is important to disentangle these two processes in emmetropic eyes and myopic eyes. Studies have demonstrated that myopic eyes exhibit a period of axial elongation rate comparable to emmetropic eyes prior to the onset of myopia; however, the process of axial elongation persists or becomes accelerated long after this process has terminated for emmetropic eyes [22,23]. In the Guangzhou Twin Registry study, it was found that annual axial elongation increased from 0.20 mm before myopia onset to 0.43 mm at the year of onset, and then reduced to 0.21 mm after the onset [6]. In our study, the annual change in ALs for grade 1 (6–9 years) was 0.39 mm, and then changed to 0.4 mm for grade 4 (9–12 years) and decreased to 0.21 mm for grade 7 (12–15 years) for myopic eyes; the growth pattern was consistent with the PNM group, but with a greater growth rate. Our data lend further support to the hypothesis that AL growth in myopic eyes is mediated by excess growth during a period in which physiological emmetropization has tapered off, a process that is not solely explained by physiological growth itself but rather by an additional myopic axial elongation pressure. A recent study showed that in emmetropia, body growth and axial elongation are correlated; however, in myopia, body growth appears to stabilize whilst axial elongation continues at a much faster rate, indicating dysregulation of normal ocular growth [14]. Furthermore, in myopes aged 12 to 22 years, AL progressed more slowly compared to those aged 6–16 years [24]. In our study, PM and PNM eyes showed similar axial elongation patterns, with rates of elongation of both groups slowing after 10 to 12 years old, which we speculate is due to the slowing of physiological growth and increases in body stature. It is unclear to us whether the underlying process of emmetropization occurs at the same time for myopic eyes compared with non-myopic eyes. It is possible that emmetropization may be completed earlier due to the compounded elongation pressures from physiological and myopic growth. On the other hand, the onset of myopia may still be occurring at any time during the emmetropization process, resulting in similar emmetropization times between PNM and PM eyes. Characterization of the exact trajectory of axial elongation in children requires further studies with a larger sample and longer follow-up periods.

Although there have been numerous cross-sectional studies or longitudinal cohort studies investigating the longitudinal changes in ocular biometry and SER among children [25], very few of them have been able to distinguish the effect of physical growth and myopia development on AL growth. Many studies do not have sufficient longitudinal follow-up for children, with an age range as wide as 3 to 15 years old. We intentionally selected the Zengcheng district for its rural setting and very low baseline prevalence of myopia; for example, myopia prevalence was 1.04% and 52.6% among 6 and 12 year olds, respectively, in Zengcheng, while these figures were 5.7% and 78.4%, respectively, for an urban population study in Guangzhou [26]. The fact that the study cohort had a very low prevalence of myopia enabled the study of sufficient numbers of children that remained emmetropic during the follow-up, and thus facilitated the investigation of AL growth not driven by myopia.

### Limitations and Strengths

There are several limitations to this study. Firstly, in order to maximize the pragmatic feasibility of the study, we selected students at four school years (G0, G1, G4 and G7), and we used a 3-year follow-up so that data could be obtained for students ranging from 3 to 15 years old. To this effect, one may argue that this design might be different to a study that enrols children at 3 years old with a 12-year follow-up period (though this second approach would also present its own limitations given the risk of increased attrition, and the possibility that the increased urbanization of the studied districts during the follow-up may also confound results). However, to the best of our understanding, our study design provides the most cost-effective way to simulate 12-year follow-up data, when the study population is very stable, and also with the least impact from a myopigenic environment. Secondly, in this study, we chose to report results based on 2-year follow-up data and therefore both cross-sectional and longitudinal data were pooled to generate descriptive data for the children aged 3 to 14 years old. Another limitation or concern to note was environmental factors/pressures that may have occurred during the lifetime of the participants (for instance, suppose that there was a trend towards increased near work at school that occurred in recent years; then, the effect of this pressure would be felt more strongly among the younger participants compared to the older participants). Fortunately, the education policy did not change greatly during recent years.

Some strengths of the study should be noted. As mentioned earlier, unlike other studies that often studied populations with a high baseline myopia prevalence, we specifically selected a district with a very low myopia prevalence, so that we would have sufficient power to detect the effect of physical development with the least interference from myopia. Secondly, in this study, we included children with a very wide range of ages, from 3 years to 15 years, and therefore were able to examine the cross-sectional and longitudinal data across a larger range of growth and development encompassing childhood and adolescence.

## 5. Conclusions

In conclusion, this longitudinal study further characterizes the AL growth driven by both physical and myopia development from 3 to 15 years. Stature growth and AL growth are both consistent; however, after 12 years old, excessive AL growth is dominated by myopia development.

## Figures and Tables

**Table 1:** Baseline distribution of SER and AL among persistent non-myopia and persistent myopia groups.

	SER (D)					AL (mm)				
	PNM		PM		*p* Value *	PNM		PM		*p* Value ^†^
	*n*	Median (IQR)	*n*	Median (IQR)		*n*	Mean (SD)	*n*	Mean (SD)	
**G0**	293	1.375 (1.125, 1.750)	1	−1.625 (−1.625, −1.625)	/	292	22.06 (0.62)	1	22.89 (/)	/
**G1**	1161	1.250 (1.000, 1.625)	11	−1.375 (−1.625, −0.750)	<0.001	1164	22.59 (0.67)	11	23.28 (0.77)	<0.001
**G4**	1041	0.875 (0.625, 1. 250)	249	−1.375 (−2.250, −0.750)	<0.001	1026	23.01 (0.72)	249	24.08 (0.77)	<0.001
**G7**	279	0.625 (0.375, 1.000)	421	−2.125 (−3.125, −1.125)	<0.001	281	23.39 (0.72)	421	24.62 (0.93)	<0.001

G0: kindergarten; G1: first year of primary school; G4: fourth year of primary school; G7: first year of junior high school; SER: spherical equivalent error; AL: axial length; PNM: persistent non-myopia; PM: persistent myopia; IQR: interquartile range; SD: standard deviation; * Wilcoxon rank sum test; ^†^ Two-sample *t* test.

**Table 2:** SER, AL and body height change during two-year follow-up.

	SER Change Rate (D/Year)				AL Change Rate (mm/Year)				Height Change Rate (cm/Year)			
	PNM		PM		PNM		PM		PNM		PM	
	*n*	Mean (SD)	*n*	Mean (SD)	*n*	Mean (SD)	*n*	Mean (SD)	*n*	Mean (SD)	*n*	Mean (SD)
**G0**	1089	0.01 (0.34)	1	/	1064	0.18 (0.09)	1	/	1091	4.54 (1.74)	1	/
**G1**	1215	−0.15 (0.19)	11	−0.72 (0.67)	1216	0.16 (0.08)	11	0.39 (0.23)	1214	4.48 (1.30)	11	4.75 (0.50)
**G4**	1088	−0.24 (0.21)	249	−0.79 (0.35)	1083	0.17 (0.09)	242	0.40 (0.15)	1085	6.13 (1.69)	244	6.16 (1.52)
**G7**	323	−0.12 (0.22)	421	−0.42 (0.26)	323	0.11 (0.08)	421	0.21 (0.11)	323	4.17 (2.85)	421	3.72 (2.68)

G0: kindergarten; G1: first year of primary school; G4: fourth year of primary school; G7: first year of junior high school; SER: spherical equivalent error; AL: axial length; PNM: persistent non-myopia; PM: persistent myopia; SD: standard deviation.

**Table 3:** Linear regression analysis for the association of the height and AL growth and SER change for PNM children.

Height Change in Different Grades	AL Growth		SER Change	
	Standardized β (95% CI)	*p*	Standardized β (95% CI)	*p*
**G0**	0.11 (−0.02, 0.23)	0.088	0.04 (−0.09, 0.16)	0.568
**G1**	0.09 (0.04, 0.15)	0.002	−0.03 (−0.09, −0.03)	0.310
**G4**	0.17 (0.11, 0.23)	<0.001	−0.14 (−0.20, −0.09)	<0.001
**G7**	0.06 (−0.06, 0.18)	0.308	−0.02 (−0.14, 0.09)	0.679

G0: kindergarten; G1: first year of primary school; G4: fourth year of primary school; G7: first year of junior high school; SER: spherical equivalent error; AL: axial length; PNM: persistent non-myopia.

## Data Availability

All the data in this study have been listed in this article (text, tables, figures and Supplementary Materials). Requests will be honored from researchers who need additional data and should be directed by email to the corresponding author.

## Supplementary Materials

The following supporting information can be downloaded at: https://www.mdpi.com/article/10.3390/jcm11133642/s1, Figure S1: Flowchart. Figure S2: Axial length (AL) and spherical equivalent refraction (SER) change in height change in tertiles from 3 to 14 years of age in persistent non-myopia. Table S1: Sample composition based on different grades. Table S2: Baseline characteristics of the participants. Table S3: AL trend across height levels by age group, among PNM participants.
