# Supplementary material for: Axial Growth Driven by Physical Development and Myopia among Children: A Two Year Cohort Study
Source: J Clin Med. 2022 Jun 23;11(13):3642. doi: 10.3390/jcm11133642 (PMC9267224; doi:10.3390/jcm11133642)
Supplement: Supplementary file 1 [file jcm-11-03642-s001.zip › jcm-1757148-supplementary.pdf]

Supplementary Tables

Table S1. Sample composition based on different conditions

Grade	Screening sample, N	Children with cycloplegic refraction, n (%)	Children with cycloplegic refraction and measure of axial length, n (%)	Children without abnormal ocular findings*
G0	1,433	1,431 (99.9)	1,406 (98.1)	1,366 (95.3)
G1	1,561	1,561 (100)	1,553 (99.5)	1,461 (93.6)
G4	2,671	2,671 (100)	2,615 (97.9)	2,333 (87.3)
G7	1,385	1,385 (100)	1,380 (99.6)	1,193 (86.1)
Total	7,050	7,048 (99.97)	6,954 (98.6)	6,353 (90.1)

* Abnormal ocular characteristics included abnormal ocular findings, astigmatism greater than 5.0D of cylinder or high myopia (SER≤-5.0D).

G0: kindergarten; G1: first year of primary school; G4: fourth year of primary school; G7: first year of junior high school.

Table S2. Baseline characteristics of the participants (n=6353)

	n (%)	Age (year)<br>Mean (SD)	SER (D)<br>Median (IQR)	AL (mm)<br>Mean (SD)
**G0**				
**Male**	706 (51.68)	3.68 (0.33)	1.375 (1.125 – 1.625)	22.29 (0.58)
**Female**	660 (48.32)	3.69 (0.36)	1.375 (1.000 – 1.750)	21.75 (0.57)
**All**	1366	3.68 (0.35)	1.375 (1.125 – 1.750)	22.04 (0.63)
**G1**				
**Male**	816 (55.85)	6.81 (0.35)	1.250 (0.875 – 1.500)	22.85 (0.63)
**Female**	645 (44.15)	6.78 (0.35)	1.250 (0.875 – 1.625)	22.32 (0.62)
**All**	1461	6.80 (0.35)	1.250 (0.875 – 1.625)	22.62 (0.68)
**G4**				
**Male**	1263 (54.14)	9.56 (0.41)	0.625 (0.125 – 1.000)	23.51 (0.79)
**Female**	1070 (45.86)	9.54 (0.40)	0.625 (-0.125 – 0.875)	23.01 (0.79)
**All**	2333	9.55 (0.41)	0.625 (0.000 – 1.000)	23.28 (0.83)
**G7**				
**Male**	651 (54.57)	12.6 (0.41)	-0.125 (-1.875 – 0.625)	24.23 (1.05)
**Female**	542 (45.43)	12.5 (0.39)	-1.000 (-2.500 – 0.125)	23.92 (0.92)
**All**	1193	12.6 (0.40)	-0.500 (-2.250 – 0.375)	24.08 (1.00)

Table S3. AL trend across height levels by age group, among PNM participants

Age (y)	Low height*		Middle height		High height		p value
	n	Mean (SD)	n	Mean (SD)	n	Mean (SD)	
**3**	72	21.96 (0.54)	70	22.10 (0.61)	57	22.16 (0.56)	0.112
**4**	276	21.99 (0.65)	398	22.19 (0.59)	276	22.25 (0.67)	<0.001
**5**	438	22.22 (0.66)	399	22.38 (0.67)	209	22.49 (0.64)	<0.001
**6**	347	22.41 (0.67)	355	22.56 (0.64)	325	22.65 (0.68)	<0.001
**7**	397	22.55 (0.65)	511	22.74 (0.63)	508	22.82 (0.67)	<0.001
**8**	452	22.78 (0.70)	348	22.89 (0.62)	273	23.03 (0.69)	<0.001
**9**	455	22.87 (0.67)	413	23.00 (0.66)	430	23.28 (0.69)	<0.001
**10**	344	23.03 (0.69)	297	23.18 (0.71)	356	23.43 (0.70)	<0.001
**11**	360	23.20 (0.74)	289	23.36 (0.71)	340	23.52 (0.73)	<0.001
**12**	114	23.21 (0.68)	103	23.42 (0.76)	131	23.61 (0.73)	<0.001
**13**	80	23.41 (0.97)	85	23.26 (0.74)	120	23.70 (0.64)	<0.001
**14**	124	23.41 (0.64)	113	23.64 (0.79)	91	23.80 (0.70)	<0.001
**15**	22	23.57 (0.84)	18	23.59 (0.59)	2	24.07 (0.01)	0.684

*: Divided into tertiles based on body height;

PNM: persistent non-myopia; p value: AL change among the three different body height tertiles.

Supplement Figures

Figure S1 Flowchart

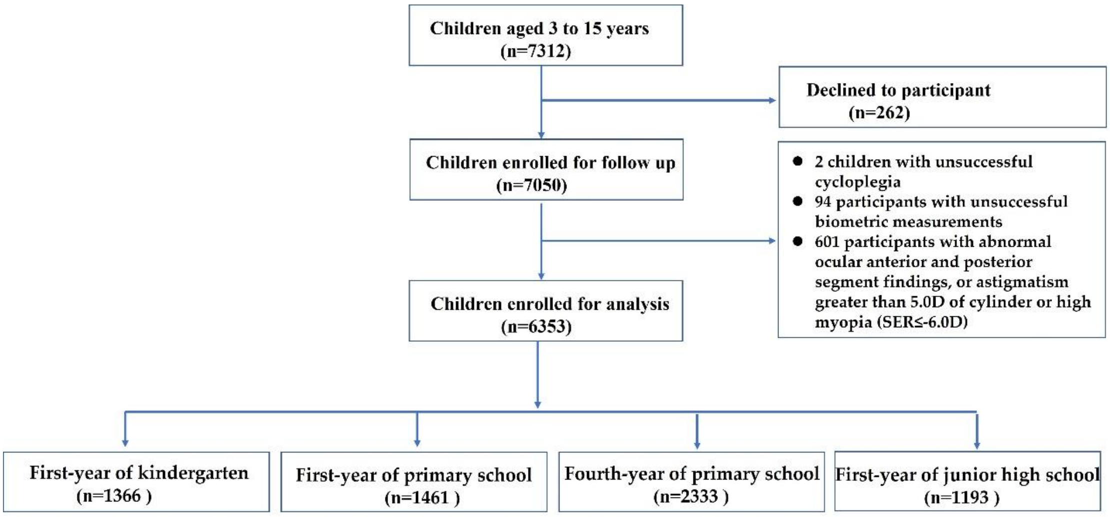

Figure S2

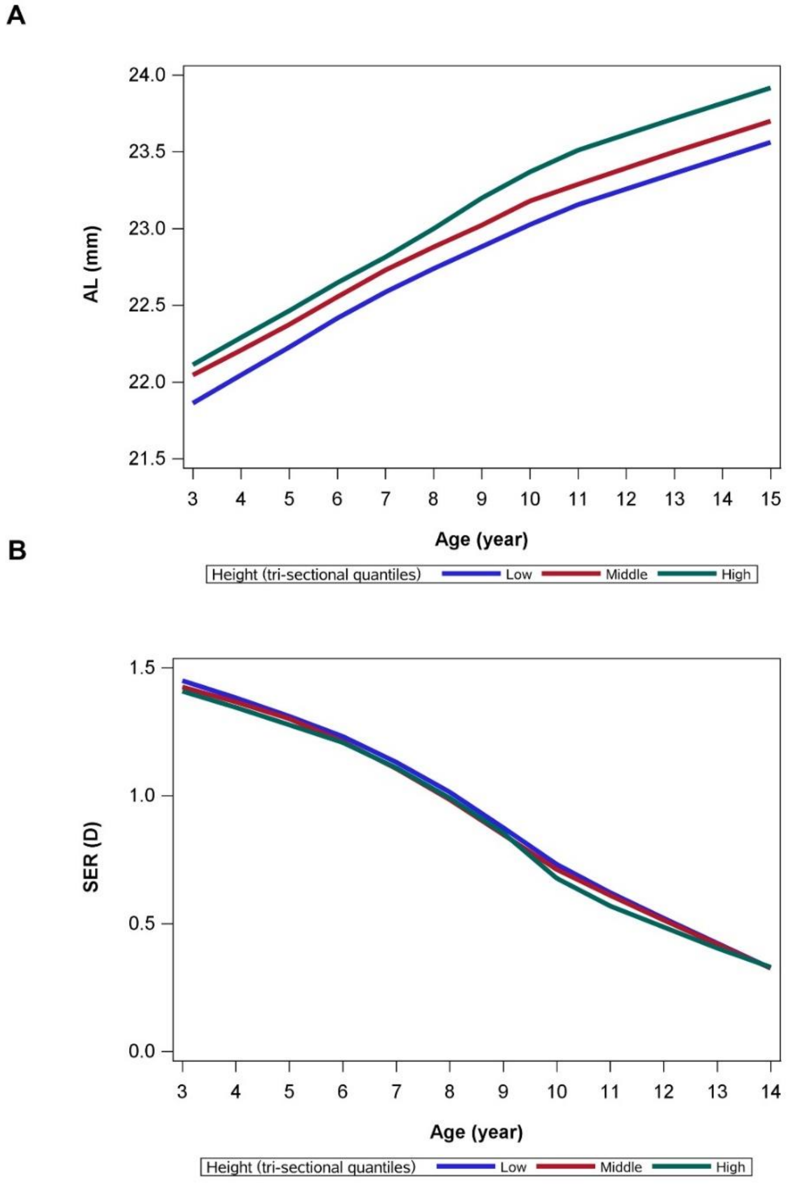

Figure S2 Axial length (AL) and spherical equivalent refraction (SER) change in height change in tertiles from 3 to 14 years of age in persistent non-myopia. A: the change of AL. B: the change of SER. Data were plotted pooling all the participants with two-year follow up.
